# Genetic Variation in the Chemokine Network and Atherosclerosis Risk

**DOI:** 10.1007/s11883-026-01401-7

**Published:** 2026-03-13

**Authors:** Panagiotis Zangas, Marios K. Georgakis

**Affiliations:** 1 Institute for Stroke and Dementia Research (ISD), LMU University Hospital, LMU, Feodor-Lynen-Str. 17, 81377 Munich, Germany; 2 Program in Medical and Population Genetics and Cardiovascular Disease Initiative, Broad Institute of MIT and Harvard, Cambridge, MA USA; 3 Munich Cluster for Systems Neurology (SyNergy), Munich, Germany

**Keywords:** Genetic variation, Inflammation, Chemokines, Atherosclerosis, Cardiovascular disease, Genetics in drug development

## Abstract

**Purpose of Review:**

This review examines the impact of genetic variation in chemokines and their receptors on the risk of atherosclerosis and adverse cardiovascular outcomes.

**Recent Findings:**

Human genetic studies have identified associations between variants in multiple chemokines and chemokine receptor loci and atherosclerotic cardiovascular disease (ASCVD), including coronary artery disease and myocardial infarction, carotid atherosclerosis and large-artery atherosclerotic stroke, as well as peripheral artery disease. Among chemokine pathways, the most consistent evidence implicates the CCL2-CCR2, CXCL12-CXCR4, and CXCL10-CXCR3 axes. Triangulation with experimental, epidemiological, and human tissue data supports the potential of targeting these pathways to reduce vascular inflammation, promote plaque stabilization, and lower ASCVD risk.

**Summary:**

Recent proof-of-concept trials have demonstrated the efficacy of anti-inflammatory therapeutics in atherosclerosis. As the field moves towards a newer generation of atherosclerosis-specific anti-inflammatory agents, human genetic evidence supports targeting chemokine pathways, particularly those governing immune cell trafficking, as a promising therapeutic strategy. These findings provide a strong background for clinical trials assessing anti-chemokine drugs for the prevention of cardiovascular events.

**Graphical Abstract:**

Graphical overview of the role of genetic variation in the chemokine network in prioritization of drug targets for atherosclerotic cardiovascular disease. GWAS – Genome-wide association studies, LoF – Loss of function, ASCVD – atherosclerotic cardiovascular disease. Created in https://BioRender.com.

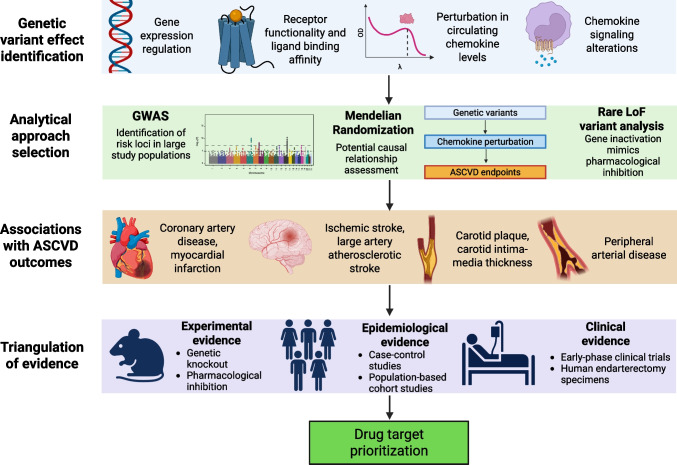

## Introduction

Atherosclerotic cardiovascular disease (ASCVD) remains the leading cause of mortality worldwide [1], and is a major contributor to disability [2]. Three decades of preclinical research have demonstrated the causal role of inflammation in atherogenesis [3, 4], and recent clinical trials investigating the efficacy of anti-inflammatory drugs, such as canakinumab and colchicine, have shown that targeting inflammatory pathways can reduce adverse cardiovascular events [5–7]. Inflammation-targeting strategies for atheroprotection must be carefully weighed against their potential side effects on host defense mechanisms. For instance, both canakinumab [5] and colchicine [6] were linked to adverse outcomes, including fatal infections.

The predominant focus of current translational efforts is on the inflammasome/IL-1β/IL-6 axis [8]. Three anti-IL6 antibodies have been proven successful in phase II studies [9–11] and are progressing into phase III cardiovascular outcome trials, and NLRP3 inflammasome antagonists are emerging as promising candidates for ASCVD treatment [12]. However, extensive experimental, epidemiological and early-phase clinical evidence highlight the potential of targeting alternative inflammatory mediators, including the chemokine system, with a more concrete role in atherosclerosis [13, 14]. Chemokines comprise a large cytokine family, which is further classified into subfamilies (CC, CXC, CX_3_C and XC) according to the position of cysteine residues at their N-terminus [15]. The chemokine network is involved in almost all stages of atherosclerosis progression [14] and exhibits various functions beyond its classical role of immune cell recruitment, including cell activation and homeostasis regulation [15]. Modulating alternative inflammatory pathways, which may be more specific to atherosclerosis, can enhance efficacy and improve safety, moving anti-inflammatory therapeutic strategies closer to successful clinical application. Several compounds targeting chemokines or their receptors have been tested in cardiovascular clinical trials, as detailed in Table 1.

**Table 1:** Drugs targeting chemokines or chemokine receptors tested in cardiovascular disease-related clinical trials

Target	Compound name	Condition	Primary study endpoint	Phase	Results	Trial ID
CCR5	Maraviroc	Atherosclerosis progression in HIV-infected patients	Change in brachial flow mediated dilation (bFMD), carotid intima-media thickness (cIMT) and carotid-femoral pulse wave velocity (cfPWV)	IV	Improvement of bFMD by 66%, cIMT by 13% and cfPWV by 11% [20]	NCT03402815
CCR2/CCR5	Cenicriviroc	Arterial inflammation in HIV-infected patients	Change (expressed as Ratio to Baseline) in 18-FDG-PET Target-to-background Ratio of the Most Diseased Segment of the index (most-inflamed) vessel	II	Completed, results not available	NCT05630885
CCR2	MLN-1202 (Plozalizumab)	Atherosclerosis	Effect on C-reactive protein (CRP)	II	Significant reduction in CRP levels [21]	NCT00715169
CCL2	Bindarit	Coronary restenosis prevention	In-segment late loss	II	No significant changes in in-segment late loss or MACE, but significant reduction in in-stent late loss [22]	NCT01269242
CXCR1/CXCR2	Reparixin	Systemic and pulmonary inflammation in CABG patients	Proportion and absolute number of neutrophils in blood and BALF	II	Significant reduction in blood neutrophil proportion, but not in absolute number. No significant alterations detected in BALF [23]	EudraCT 2004–001138-18
CXCR2	AZD5069	PCI in coronary artery disease	Change in coronary flow reserve from baseline	II	Results not available	EudraCT 2016–000775-24 (CICADA)
CXCL12	JVS-100	Ischemic heart failure	Impact on 6MWD and QoL at 4 month follow-up	II	Primary endpoint was not met [24]	NCT01643590 (STOP-HF)
CXCL12	JVS-100 (retrograde delivery)	Ischemic heart failure	Impact on 6MWD at 4 month follow-up	II	Results not available	NCT01961726 (RETRO-HF)
CXCL12	JVS-100	Critical limb ischemia	Safety and tolerability	II	Completed, results not available	NCT01410331
CXCL12	JVS-100	Peripheral arterial disease	Composite endpoint of wound progression, healing and limb loss	II	No improvement in outcomes after three [25] or six months [26]	NCT02544204 (STOP-PAD)
CXCR4	POL6326 (Balixafortide)	Myocardial infarction	Change in LVEF	II	Completed, results not available	NCT01905475 (CATCH-AMI)
CX3CR1	KAND567	ST-elevation myocardial infarction	Safety and tolerability	II	Completed, results not available	ISRCTN18402242 (FRACTAL)

Trials were identified through searches in MEDLINE and the clinicaltrials.gov website. *HIV* human immunodeficiency virus, *FDG-PET* fluorodeoxyglucose positron emission tomography, *CABG* coronary artery bypass grafting, *BALF* bronchoalveolar lavage fluid, *PCI* percutaneous coronary intervention, *6MWD* 6-min walk distance, *QoL* quality of life, *LVEF* left ventricular ejection fraction

Human genetics can provide causal insights into disease-predisposing mechanisms, thereby highlighting potential therapeutic targets [16] and guiding drug development efforts [17]. Genetically supported drug targets are approximately 2–3 times more likely to result in approved drugs [18]. This paradigm has been particularly influential in ASCVD, where several recently approved drugs or agents in advanced clinical development were initiated or strongly motivated by human genetic discoveries, including PCSK9, ANGPTL3, APOC3, LPA, FXI, and IL6 [19]. Here, we review the existing literature aiming to investigate the extent to which genetic variation in key chemokines and their receptors influences the risk of atherosclerosis and its clinical manifestations.

### The CCL2 – CCR2 axis

CCL2, also called monocyte chemoattractant protein-1 (MCP-1), is the most extensively studied chemokine [27] and is primarily responsible for chemoattraction of monocytes from the bone marrow to circulation and inflammation sites through a CCR2 (its main receptor)- dependent process [28].

Mendelian Randomization (MR) leverages naturally occurring genetic variation to assess potential causal effects of modifiable risk factors on disease-related outcomes in observational studies, accounting for presence of confounders and reverse causation [29]**.** In a MR study exploring genetic associations between 41 cytokines and risk of stroke, genetically proxied higher circulating CCL2 levels were associated with increased risk of all stroke, ischemic stroke, large-artery atherosclerotic stroke (LAAS) and cardioembolic stroke, but not small-vessel stroke and intracerebral hemorrhage [30]. The findings remained consistent across alternative MR methods and sensitivity analyses and retained statistical significance after adjusting for cardiovascular risk factors in multivariable MR [30]. Further associations were observed between genetically determined higher CCL2 levels and increased risk of CAD and MI, as etiologically linked endpoints [30]. Later MR studies replicated the genetic association between CCL2 levels and LAAS [31] and provided suggestive evidence of association between CCL2 levels and risk of stroke of undetermined source [32], which may be caused by vulnerable atherosclerotic plaques not reaching the stenosis grade needed to be classified as LAAS [33].

Naturally occurring human loss-of-function variants serve as in vivo models of gene inactivation that can imitate the effects of pharmacological inhibition and thereby guide drug target evaluation [34]. A recent study demonstrated that carriers of 45 rare damaging missense or loss-of-function *CCR2* variants were at lower risk of CAD and MI compared to non-carriers [35]. The most frequent of these variants, M249K, was additionally associated with lower monocyte count and attenuated CCL2-induced signaling and chemotaxis. M249K was consistently associated with lower risk of CAD and MI, without increasing infection risk among its carriers. Notably, the absence of differences in LDL cholesterol, blood pressure, BMI, HbA1c, and C-reactive protein (CRP) levels among M249K carriers indicates that the effects of damaging *CCR2* variants are independent of conventional cardiovascular risk factors targeted by existing atheroprotective therapies [35]. Similar efforts have been pursued for *CCL2* variants with mixing results. Although no loss-of-function variant with sufficient frequency to be studied in epidemiological studies has been identified, a promoter SNP influencing *CCL2* expression (−2518A/G, or rs1024611) was already reported in 1999 [36]. This variant, along with *CCR2* V64I (rs1799864), were proposed to be associated with CAD risk [37], but later meta-analyses showed conflicting results regarding clinical ASCVD endpoints [38–44]. Moreover, rs1799864 was not associated with CAD or MI in large-scale datasets [45].

A polygenic risk score (PRS) comprising variants in the *CCL2*, *CCR2*, and *SELE* (E-selectin) loci was associated with ischemic stroke risk, and the combination of a high PRS and low estradiol levels further increased this risk in the overall cohort and among men, but not in women, likely due to the comparatively smaller female sample size [46]. This observation is consistent with data suggesting that estrogen may modulate the CCL2-CCR2 pathway activity and monocyte chemotaxis in atherosclerosis [47].

In addition to genetic data, a solid body of evidence, consisting of experimental, epidemiological and early-phase clinical findings emphasizes the role of the CCL2-CCR2 axis as a key driver of atherosclerosis and supports the therapeutic potential of targeting its components [28]. *Ccl2* or *Ccr2* deletion led to attenuation [48–50], while *Ccl2* overexpression to acceleration of atherosclerosis progression in atheroprone mice [51]. Moreover, a meta-analysis of preclinical studies showed that pharmacological inhibition of the CCL2-CCR2 axis resulted in reduced atherosclerosis burden in atherosclerosis-prone mice [52]. Evidence from prospective observational studies indicated associations of higher circulating CCL2 levels with any or ischemic stroke [53] and cardiovascular death [54]. CCL2 levels were additionally associated with histopathologic and molecular carotid plaque vulnerability features, and were higher in symptomatic plaques compared to asymptomatic [55].

To date, two early-phase clinical trials targeting the CCL2-CCR2 axis in the context of ASCVD have been conducted (Table 1). A phase II randomized placebo-controlled trial (RCT) of the CCR2-inhibiting antibody MLN1202 demonstrated significant decreases in CRP levels in patients with at least two cardiovascular risk factors [21], while a phase II RCT of the non-specific CCL2 inhibitor bindarit failed to meet the primary endpoint of in-segment late loss in coronary stenting patients, despite achieving the secondary endpoint of in-stent late loss [22]. Interestingly, in the MLN1202 trial, among the carriers of the G allele of the above mentioned SNP rs1024611, these treated with MLN1202 experienced significantly higher decreases in CRP compared to placebo, while no significant differences were observed between the two groups among participants homozygous for the A allele [21]. To our knowledge, none of the two compounds has progressed into a phase III RCT.

### The CXCL12 – CXCR4 Axis

The chemokine CXCL12, or stromal cell-derived factor 1α, is responsible for homing of progenitor cells to the bone marrow and mobilizing them to the periphery in situations of tissue injury or stress, acting mainly on its receptor CXCR4 [56]. Alongside the CCL2-CCR2 axis, the CXCL12-CXCR4 axis exhibits the strongest genetic association evidence in the context of ASCVD. Multiple genetic variants in the *CXCL12* locus were associated with CAD and MI in the largest genome-wide association studies (GWAS) to date, both meta-analyses of the CARDIoGRAMplusC4D consortium and the UK Biobank [57, 58]. In a smaller GWAS meta-analysis, the intergenic SNP rs2802492 near *CXCL12* was linked to both CXCL12 levels and risk of CAD [59], while further associations were observed between variants close to *CXCL12*, CXCL12 plasma levels [60] as well as *CXCL12* expression in human coronary and tibial arteries [61]. Additionally, a MR analysis showed associations between genetically proxied elevation of CXCL12 levels and increased CAD/MI risk [62]. An epidemiologic analysis in the same cohort demonstrated an association between elevated CXCL12 levels and major adverse cardiovascular events (MACE) [62].

The *CXCR4* locus SNP rs4954580 reached genome-wide significance after a meta-analysis of the aforementioned CAD GWAS with Biobank Japan [57]. Moreover, the A and C alleles of the *CXCR4* locus SNPs rs2228014 and rs2322864, respectively, were associated with increased CAD risk [63, 64], with the latter being also linked to reduced *CXCR4* expression in a whole blood expressive quantitative trait loci (eQTL) analysis [63]. The CC genotype of this SNP was connected to reduced *CXCR4* mRNA expression in human atherosclerotic plaques, and *CXCR4* expression was lower in symptomatic plaques compared to asymptomatic [63].

The role of the CXCL12-CXCR4 axis in atherosclerosis is equivocal. An atheroprotective, plaque-stabilizing role of CXCL12 in atheroprone mice has been suggested, attributed to enhanced recruitment of smooth muscle cell (SMC) progenitor cells [65], function which also improved atherosclerotic lesion stability after transient CXCL12 injections [66]. Disruption of the CXCL12-CXCR4 axis through receptor inhibition with the CXCR4 antagonist AMD3465 or deficiency in bone marrow *Cxcr4* aggravated atherosclerosis in mice [67]. On the other hand, high CXCL12 expression levels were observed in SMCs, endothelial cells and macrophages in atherosclerotic plaques, in contrast to healthy vessels [68], and CXCL12 synthesis by endothelial cells has been recently identified as a key mediator of atherosclerosis and contributor to circulating CXCL12 levels [59]. Furthermore, plasma CXCL12 levels are an independent predictor of adverse cardiovascular outcomes in CAD patients [69].

With respect to cardiovascular clinical trials targeting the components of the axis, a non-viral, naked DNA plasmid encoding human CXCL12 (JVS-100) was developed to target chronic ischemic heart failure and critical limb ischemia/peripheral arterial disease (PAD). Of the four registered phase II RCTs (Table 1), two have reported results, demonstrating that intramyocardial (for heart failure) or intramuscular (for PAD) JVS-100 injection did not significantly improve endpoints compared with placebo [24–26]. POL6326 (balixafortide), a CXCR4 antagonist, completed a phase II RCT evaluating its effect on left ventricular ejection fraction and other measures of cardiovascular function in patients with acute MI, but the results are not yet available (Table 1).

### The CXCL10 - CXCR3 Axis

CXCL10, or interferon-inducible protein-10, is secreted mainly upon interferon-γ production and induces its effects by binding to CXCR3[70]. Its mechanism of action is complex and depends on the CXCR3 isoform it acts on, ranging from leukocyte chemotaxis (mainly Th1 lymphocytes), endothelial cell and vascular SMC migration and proliferation through the CXCR3-A isoform to antiangiogenic and pro-apoptotic effects through the CXCR3-B isoform [70]. A multifaceted proteogenomic analysis demonstrated that genetically proxied CXCL10 levels were associated with risk of CAD, LAAS and PAD, and changes in CXCL10 levels mediated a substantial proportion of the IL-6 signaling effects on these endpoints [71]. This study additionally demonstrated that elevated circulating midlife CXCL10 levels were linked to increased cardiovascular event occurrence over 20 years, whereas elevated CXCL10 expression in human atherosclerotic lesions correlated with a larger lipid core and a transcriptomic signature indicative of inflammatory cell infiltration, adaptive immune activation, and cytokine signaling [71]. Another study showed that serum CXCL10 levels were higher and the GG genotype of the *CXCL10* promoter SNP rs4508917 was more frequent in ischemic heart disease (IHD) patients than controls, and IHD patients with GG genotype had higher CXCL10 levels than patients with AG or AA, though this difference was not significant [72].

Complementing the genetic evidence, both animal and human studies have demonstrated the involvement of the CXCL10-CXCL3 axis in initiation and progression of atherosclerosis. In atheroprone murine models, direct antibody-mediated CXCL10 inhibition promoted plaque stabilization [73], whereas CXCR3 blockade suppressed atherogenesis [74, 75]. Furthermore, *Cxcl10* or *Cxcr3* deletion resulted in attenuated atherogenesis and elevated lesional numbers of regulatory T cells [76, 77]. In human carotid endarterectomy specimens, higher CXCL10 levels were linked to a vulnerable plaque phenotype, characterized by macrophage infiltration, fewer SMCs and less collagen [73], while circulating CXCL10 levels were associated to CAD risk [78] and coronary atherosclerosis severity, as defined by the Gensini score [79].

### The CCL5 - CCR5 Axis

CCL5, or RANTES (regulated on activation normal T cell expressed and secreted) exerts its proatherogenic effects primarily through its main receptor CCR5. The CCL5-CCR5 axis is substantial for monocyte recruitment [80] and vascular remodeling [81] in atherosclerosis. The well-studied CCR5 deleterious variant CCR5Δ32 (rs333) and the CCL5 promoter SNP −403G/A (rs2107538) were associated in several studies with ASCVD endpoints [82], results from meta-analyses were though contradictory [83–86]. Similar to *CCR2* V64I, no *CCL5* or *CCR5* SNPs were associated with CAD or MI in large-scale GWAS or exome sequencing studies [45, 87]. Findings from MR studies are also conflicting, with genetically proxied increases in CCL5 levels being associated with increased risk of ischemic and small-vessel stroke [31], but decreased risk of LAAS [88].

In experimental studies, inhibition of the CCL5-CCR5 axis through either gene deletion or antagonism of each of the two molecules attenuated atherosclerosis in mouse models [82]. High CCL5 levels in carotid plaques were also associated with unstable plaque phenotype, despite the null effect on coronary events [87]. Furthermore, the CCR5 antagonist maraviroc, approved for HIV infection treatment [82], reduced atherosclerosis progression in both mouse [89] and human subjects [20] (Table 1).

### The CXCL8 – CXCR2 Pathway

CXCL8, commonly known as interleukin-8, was the first chemokine ever discovered and was described initially as a monocyte-derived neutrophil chemotactic factor [90]. Its role in atherosclerosis is characterized additionally by monocyte adhesion, promotion of angiogenesis, migration and proliferation of vascular SMCs [91]. Meta-analyses have shown an association of the CXCL8 promoter SNP rs4043 with increased CAD risk in total and Asian study population, but interestingly, with no association or even decreased risk in Caucasians [92, 93]. Another study showed a positive correlation between plasma CXCL8 levels and cIMT, and the association of two SNPs (rs117518778 and rs8057084, both outside the *CXCL8* locus) with both CXCL8 levels and cIMT measurements [94], while genetically proxied CXCL8 levels were associated with LAAS risk in a MR study [95]. Moreover, the C allele of the *CXCR2* (one of the receptors CXCL8 binds on) SNP rs1126579 was associated with increased risk of stroke in heterozygous as well as decreased risk in homozygous hypertensive individuals [96], although another study showed no association of the same SNP with stroke onset or recurrence [97].

The administration of CXCL8-targeting antibodies attenuated inflammation and increased stability of atherosclerotic plaques in an animal study [98], whereas human studies exploring associations between circulating CXCL8 levels and ASCVD have shown inconsistent results [94]. AZD5069, an CXCR2 inhibitor, has been tested in a phase II clinical trial in CAD patients undergoing percutaneous coronary intervention (PCI) [99], with the trial results not being currently available (Table 1).

### The CX3CL1 – CX3CR1 Pathway

CX_3_CL1, also called fractalkine, is the only known chemokine containing the CX_3_CL1 motif, and exists as both membrane-bound and soluble form [100, 101]. The membrane-bound form is strongly induced by inflammatory cytokines on activated primary endothelial cells, promotes firm adhesion of monocytes and T lymphocytes. The soluble form is released by proteolysis of the membrane-bound form, and acts as a chemoattractant for these cell types [100, 101]. CX_3_CL1 binds with high affinity to its receptor CX_3_CR1, triggering intracellular signaling and modulating monocyte adhesion [100]. Both *Cx3cr1* deletion [102, 103] and pharmacological targeting of the receptor [104, 105] had atheroprotective effects on mice. Interestingly, combined deletion of *Cx3cr1* and the aforementioned *Ccl2* and *Ccr5* nearly eliminates atherosclerosis in hypercholesterolemic, atheroprone mice [106]. CX3CR1 was also expressed in foam cells and SMCs of human atherosclerotic coronary arteries, but not in normal vessels [107].

In the early 2000 s, two *CX3CR1* coding region SNPs, T280M (rs3732378) and V249I (rs3732379), common in Caucasians and in complete linkage disequilibrium with each other, were linked to reduced CX_3_CL1 binding affinity and faster progression to AIDS in HIV-positive patients [108]. The two SNPs were additionally found to be associated with reduced ASCVD risk [109–111], but the findings of later studies were inconsistent [112]. In a meta-analysis in Caucasian population, the 280 M allele heterozygosity and the I_249_M_280_ haplotype were associated with lower risk of CAD [112]. These results were replicated in a larger cross-ancestry meta-analysis, which additionally demonstrated associations between the 249I allele heterozygosity and reduced CAD risk, the 280 M homozygosity and increased risk of ischemic cerebrovascular disease and the VITM/IITM genotypes with reduced atherosclerosis risk compared to the VVTT genotype [113]. These findings were however accompanied by significant heterogeneity across the included studies [113]. Furthermore, the two SNPs were not associated with CAD or MI in large-scale genomic datasets, with the exception of an association of the V249I variant with MI in a South Asian population, which was not replicated in the predominantly European CARDIoGRAMplusC4D consortium GWAS meta-analysis [45]. Notably, a recent MR study showed an inverse correlation between CX_3_CL1 levels and PAD risk [114].

### Other Chemokines

The chemokine CXCL16 functions as a scavenger receptor for oxidized low-density lipoprotein, and serves as an adhesion molecule and chemoattractant for cells expressing its sole receptor CXCR6 [115]. The *CXCL16* missense variant A181V (rs2277680) was linked to increased coronary artery stenosis in post-MI patients [116], however further studies of this SNP and others in the *CXCL16* region showed conflicting results regarding ASCVD phenotypes [115, 117–121].

The −1382A/G variant (rs4795895) in the promoter of the gene coding for CCL11 (eotaxin-1) was associated with ischemic stroke and its subtypes [122–124], including intracranial LAAS [123, 124]. A *CCL11* threonine for alanine substitution (rs1129844) was first described as a variant related to increased MI risk [125], but the findings of later studies with ASCVD endpoints were ambiguous [126–132]. Lastly, the *CCL11* promoter SNP rs17809012 was associated with LAAS [132] and event-free survival in CAD patients [126]. Further associations between variants related to chemokines or their receptors and ASCVD outcomes are detailed in Table 2.

**Table 2:** Associations between chemokine and chemokine receptor-related variants and ASCVD outcomes

Chemokine/receptor	SNP	Description	References
CCL7	rs17735770	Association of C allele with decreased CAD susceptibility	[133]
CCL15	rs28929474 (trans-pQTL)	MR association with reduced CAD risk	[134]
CCL3	−906 T/A	Association with internal carotid stenosis and plaque instability	[135]
CXCL5	rs352046	Association of C allele with increased CXCL5 plasma levels and CAD risk	[136]
CXCL1	rs3117604	Association of T allele with increased ischemic stroke risk	[137]
CCL27	rs2070074	Genetically predicted higher CCL27 levels have a protective effect on subsequent MACE after stroke	[138]
CCL22	rs4359426	Association of the AA genotype with lower IHD risk	[72]
CCL19	rs2227302	Association of the T allele with higher plasma CCL19 levels and increased CAD risk	[139]
CCL21	rs2812377	Association of the G allele with higher plasma CCL21 levels and increased CAD risk	[139]
CCR7	rs588019	Association of the A allele with increased CAD risk	[139]
CCR7	rs17708087	Association of the A allele with previous MI in CAD patients	[140]
CCL17	rs223828	Association of the T allele with increased CAD risk, higher serum CCL17 levels and enhanced *CCL17* promoter activity	[141]
CCL17	rs223899	Association of the T allele with increased CAD risk	[141]

*CAD* coronary artery disease, *pQTL* protein quantitative trait loci, *MR* Mendelian randomization, *CAD* coronary artery disease, *MACE* major adverse cardiovascular events, *IHD* ischemic heart disease, *MI* myocardial infarction

## Conclusions

Chemokines and their receptors can act synergistically, exerting various effects besides recruiting inflammatory cells from the bloodstream to sites of inflammation, and their expression and function is often dysregulated in ASCVD. Several therapeutic strategies targeting the chemokine system have been developed and tested in preclinical and early-phase clinical studies, although successful translation into human atherosclerotic disease remains challenging and requires further investigation [13, 14]. The limited ability of preclinical disease models to accurately predict therapeutic benefit in humans is a central problem in drug discovery, resulting in failure of a high proportion of drug development efforts [142]. Moreover, no chemokine-targeting agents have been tested in phase 3 cardiovascular outcome trials, while the current evidence from early-phase trials remains inconclusive. The fact that chemokine-targeting therapeutics have not reached advanced stages of clinical development may be attributed to challenges such as suboptimal target and dosage selection, inappropriate selection of efficacy endpoints, incomplete understanding of target biology, and the complexity of the chemokine network [143]. This highlights the need for further optimization of current chemokine-targeting strategies to enhance precision, specificity and potential for therapeutic efficacy in the context of atherosclerosis.

The chemokine network may be indirectly impacted by approved lipid-lowering and anti-inflammatory drugs for cardiovascular risk reduction. The strongest, supported by clinical trials, evidence for chemokine modulation exists for statins and colchicine. Statins have been reported to affect multiple components of the chemokine network in CAD patients [144], and they are proposed to exhibit part of their pleiotropic effects on ASCVD through downregulation of the CCL2-CCR2 axis [145]. However, in the A to Z (Aggrastat to Zocor) trial, statins had only a modest effect on CCL2 levels, and CCL2 did not serve as a reliable marker for identifying patients who could gain additional benefit from intensive statin therapy following an acute coronary syndrome [146]. Further evidence is thus required to clarify the extent to which the inhibition of the CCL2-CCR2 pathway contributes to the atheroprotective effect of statins [145]. Colchicine administration in acute coronary syndrome patients resulted in reduced transcoronary levels of CCL2, CCL5 and CX_3_CL1 compared to patients receiving no treatment [147]. Therefore, emerging direct chemokine-targeting strategies will need to demonstrate additive clinical benefit beyond current standard-of-care medications for ASCVD to justify their translational and regulatory advancement.

Human genetics have complemented decades of experimental and epidemiological evidence, strengthening support for the involvement of the chemokine system in the pathogenesis of atherosclerosis. Key genetic variants affecting chemokine expression levels or receptor functionality have been identified and associated with ASCVD endpoints and subclinical atherosclerosis markers in case–control and cohort studies. Nevertheless, such study types are characterized by relatively small sample sizes, limiting statistical power, and meta-analyses combining different ethnic populations showed inconsistent results, accompanied by high heterogeneity and ancestry-related differences in several studies. The restricted generalizability across diverse ancestral groups is a key limitation of genetic association studies. Most large-scale GWAS commonly used as data sources for MR analyses consist primarily of European populations. In contrary, studies representing non-European populations often lack sufficient statistical power to detect significant associations, largely because of their smaller sample sizes. Furthermore, despite the availability of large-sample GWAS for CAD [57], MI [58] and other ASCVD endpoints, genome-wide significant associations were observed only with variants in the *CXCL12* and *CXCR4* loci, which are not located in coding regions of the respective genes, highlighting the need for clarifying their action mechanism in the pathogenesis of CAD and MI.

Drug target MR analyses have shown strong concordance with clinical trial outcomes across a broad spectrum of diseases, including ASCVD, underscoring their value for supporting therapeutic target validation [148]. MR studies have identified associations between genetically proxied levels of multiple chemokines and ASCVD outcomes, with the strongest evidence, consistent in epidemiological analyses, observed for the CCL2-CCR2 [30], CXCL12-CXCR4 [62] and CXCL10-CXCR3 [71] axes. A major advantage of MR is that it assesses the cumulative effect of the SNPs on the trait of interest, in contrast to analyses using individual variants, whose effects on the trait are considered generally modest. On the other hand, microarray-based GWAS, utilized frequently in MR, usually do not include rare variants which could have a meaningful impact on ASCVD endpoints, such as the *CCR2* M249K variant [35].

In summary, human genomic evidence supports a putative causal role of several chemokine pathways in atherosclerosis development and progression, providing a rationale for prioritizing specific chemokines and their receptors for ASCVD clinical trials. A deeper understanding of how genetic variation in chemokines influences immune-vascular interactions is essential for translating genetic insights into targeted strategies for atherosclerosis prevention and treatment.

## Key References

- Georgakis MK, Bernhagen J, Heitman LH, Weber C, Dichgans M. Targeting the CCL2–CCR2 axis for atheroprotection. Eur Heart J. 2022;43:1799–808. 10.1093/eurheartj/ehac094.
  - ○ Recent comprehensive review summarizing experimental, epidemiological, genetic and clinical findings with respect to the role of the CCL2-CCR2 axis in atherosclerosis and the therapeutic potential of its targeting.
- Georgakis MK, Gill D, Rannikmäe K, Traylor M, Anderson CD, MEGASTROKE consortium of the International Stroke Genetics Consortium (ISGC), et al. Genetically Determined Levels of Circulating Cytokines and Risk of Stroke. Circulation. American Heart Association; 2019;139:256–68. 10.1161/CIRCULATIONAHA.118.035905.
  - ○ Mendelian Randomization study demonstrating an association between genetically proxied circulating CCL2 levels and risk of stroke, which was confirmed in a meta-analysis of observational studies. Further associations were observed with ischemic, cardioembolic and large artery atherosclerotic stroke, as well as CAD and MI.
- Georgakis MK, Malik R, Bounkari OE, Hasbani NR, Li J, Huffman JE, et al. Rare damaging CCR2 variants are associated with lower lifetime cardiovascular risk. Genome Medicine. 2025;17:27. 10.1186/s13073-025-01456-2.
  - ○ Rare variant analysis demonstrating the association between rare damaging *CCR2* variants and lower lifetime risk of CAD and MI, providing genetic support for the translational potential of pharmacological CCR2 targeting for atherosclerosis risk reduction.
- Aragam KG, Jiang T, Goel A, Kanoni S, Wolford BN, Atri DS, et al. Discovery and systematic characterization of risk variants and genes for coronary artery disease in over a million participants. Nat Genet. Nature Publishing Group; 2022;54:1803–15. 10.1038/s41588-022-01233-6.
  - ○ The largest CAD GWAS published to date, including more than one million participants. This analysis identified > 250 risk loci for CAD, among them *CXCL12* and *CXCR4.*
- Hartiala JA, Han Y, Jia Q, Hilser JR, Huang P, Gukasyan J, et al. Genome-wide analysis identifies novel susceptibility loci for myocardial infarction. Eur Heart J. 2021;42:919–33. 10.1093/eurheartj/ehaa1040.
  - ○ The largest MI GWAS currently conducted, consisting of > 600,000 participants. *CXCL12* was among the 80 genomic loci significantly associated with MI risk.
- Sjaarda J, Gerstein H, Chong M, Yusuf S, Meyre D, Anand SS, et al. Blood CSF1 and CXCL12 as Causal Mediators of Coronary Artery Disease. JACC. American College of Cardiology Foundation; 2018;72:300–10. 10.1016/j.jacc.2018.04.067.
  - ○ Mendelian Randomization study showing associations between genetically proxied elevated CXCL12 levels and increased risk of CAD and MI. Higher circulating CXCL12 levels were additionally associated with MACE in an epidemiological analysis.
- Prapiadou S, Živković L, Thorand B, George MJ, van der Laan SW, Malik R, et al. Proteogenomic Data Integration Reveals CXCL10 as a Potentially Downstream Causal Mediator for IL-6 Signaling on Atherosclerosis. Circulation. American Heart Association; 2024;149:669–83. 10.1161/CIRCULATIONAHA.123.064974.
  - ○ A multifaceted proteogenomic analysis which identified associations between CXCL10 levels and multiple ASCVD phenotypes (CAD, LAAS, PAD). CXCL10 mediated a significant proportion of the effect of IL-6 signaling on these outcomes. These findings were supported by epidemiological, histological and transcriptomic data.

## Data Availability

No datasets were generated or analysed during the current study.
